# Patterns of soil-transmitted helminth infection and impact of four-monthly albendazole treatments in preschool children from semi-urban communities in Nigeria: a double-blind placebo-controlled randomised trial

**DOI:** 10.1186/1471-2334-9-20

**Published:** 2009-02-19

**Authors:** Patrick Kirwan, Samuel O Asaolu, Síle F Molloy, Titilayo C Abiona, Andrew L Jackson, Celia V Holland

**Affiliations:** 1 Department of Zoology, University of Dublin, Trinity College, Dublin 2, Ireland; 2 Department of Zoology, Obafemi Awolowo University, Ile-Ife, Nigeria; 3 HIV/AIDS Research and Policy Institute, Chicago State University, Chicago, IL, USA

## Abstract

**Background:**

Children aged between one and five years are particularly vulnerable to disease caused by soil-transmitted helminths (STH). Periodic deworming has been shown to improve growth, micronutrient status (iron and vitamin A), and motor and language development in preschool children and justifies the inclusion of this age group in deworming programmes. Our objectives were to describe the prevalence and intensity of STH infection and to investigate the effectiveness of repeated four-monthly albendazole treatments on STH infection in children aged one to four years.

**Methods:**

The study was carried out in four semi-urban villages situated near Ile-Ife, Osun State, Nigeria. The study was a double-blind placebo-controlled randomised trial. Children aged one to four years were randomly assigned to receive either albendazole or placebo every four months for 12 months with a follow-up at 14 months.

**Results:**

The results presented here revealed that 50% of the preschool children in these semi-urban communities were infected by one or more helminths, the most prevalent STH being *Ascaris lumbricoides *(47.6%). Our study demonstrated that repeated four-monthly anthelminthic treatments with albendazole were successful in reducing prevalence and intensity of *A. lumbricoides *infections. At the end of the follow-up period, 12% and 43% of the children were infected with *A. lumbricoides *and mean epg was 117 (S.E. 50) and 1740 (S.E. 291) in the treatment and placebo groups respectively compared to 45% and 45% of the children being infected with *Ascaris *and mean epg being 1095 (S.E. 237) and 1126 (S.E. 182) in the treatment and placebo group respectively at baseline.

**Conclusion:**

Results from this study show that the moderate prevalence and low intensity of STH infection in these preschool children necessitates systematic treatment of the children in child health programmes.

**Trial Registration:**

Current controlled trials ISRCTN44215995.

## Background

Soil-transmitted helminths (STH) are included in the list of the world's neglected tropical diseases [1]. The STHs include the roundworm *Ascaris lumbricoides*, the whipworm *Trichuris trichiura*, the hookworms, *Ancylostoma duodenale *and *Necator americanus*, and *Strongyloides stercoralis*. The global burden of disease caused by these intestinal nematodes is an estimated 22.1 million disability adjusted life years (DALYs) lost to hookworm, 10.5 million to *A. lumbricoides*, 6.4 million to *T. trichiura*, giving a combined total of 39 million life years [2]. The wider community has recognised the importance of STHs acknowledging that their disease burden is as great as those of tuberculosis (34.7 million DALYs) or malaria (46.5 million DALYs) [3].

Growth stunting, iron-deficiency anaemia, rectal prolapse and chronic dysentery are features of STH infections; these parasitic infections can also adversely affect cognitive development in childhood [4]. The morbidity due to STH infections is greatest in school-age children who typically have the highest intensity of helminth infections [5-10]. In 2001, the World Health Assembly passed a resolution urging member states to control the morbidity of STH infections through large-scale use of anthelminthic drugs for school-aged children in less developed countries [11]. Children aged between one and five years are particularly vulnerable to disease caused by STH infections [4,12,13]. Although, children of these age groups are less likely to harbour heavy infections, their worm burdens are housed in smaller bodies, and therefore they are at a higher risk of anaemia and wasting malnutrition [14].

Preschool children, defined as aged less than five years, make up between 10%–20% of the two billion people worldwide who are infected with STHs; 21 million preschool children are infected with hookworm, 122 million are infected with *A. lumbricoides *and 86 million are infected with *T. trichiura *[15]. Periodic deworming has been shown to improve growth, micronutrient status (iron and vitamin A), and motor and language development in preschool children and justifies the inclusion of this age group in these deworming programmes [16]. Further assessment of the drug efficacy of anthelminthic drugs in this age group is needed [16] with particular attention to the optimal dosage of anthelminthics in order to maximise the efficacy and minimise the risks of these drugs in children aged less than 24 months [17].

This study was undertaken as part of a larger study investigating the relationship between STHs and malaria in preschool children. Our objectives were to describe the prevalence and intensity of STH infection and to investigate the effectiveness of repeated four-monthly albendazole treatments on STH infection in children aged one to four years.

## Methods

### Study area and participants

The study was carried out between May 16, 2006, and August 22, 2007, in four semi-urban villages, Akinlalu, Ipetumodu, Moro and Edunabon, situated near Ile-Ife, Osun State, Nigeria. The climate is characterised by a high uniform temperature, moderate to heavy seasonal rainfall, and high relative humidity. The dry season extends from November to March while the rainy season occurs from April to October [18]. Annual rainfall in the region ranges from 1000 to 4000 mm [18]. The average maximum and minimum daily temperatures are 32°C and 20°C respectively and the vegetation is rainforest[18].

The inhabitants of these communities are a mixture of people from different ethnic groups, although the majority are Yoruba-speaking. Houses in the villages are predominately built of concrete floors and walls and roofed with corrugated galvanised iron sheets. There is no organised sewage disposal system and refuse and human faeces are dumped in the bush or burned. The main source of water is from shared community taps and/or wells located in each village. Health care is provided in health centers located in each village, these are inadequately equipped and lack essential supplies and qualified staff. STHs are endemic in this region [9,18].

Meetings were held with the Obas (traditional head of a Yoruba village), Ife North Local Government and inhabitants of the village in May 2006 to explain the aims of the study. The Obas made a call for children aged 12–60 months to attend temporary clinics for assessments on specified dates. The assessments took place in the town hall at the Oba's palace, which was located at the centre of each village. Participation in the study was voluntary. The study was explained to each mother on the day of the assessment and they were asked to sign or finger print the consent form to enroll their child. The consent forms were provided in English and Yoruba. Each child was given an identification card with an ID number and was requested to present this card on all subsequent visits. Mothers were interviewed using a questionnaire by a trained fieldworker who collected data on age and gender of the child and socio-economic status. The study protocol was approved by the Ethics and Research Committee, Obafemi Awolowo University Teaching Hospitals' Complex, Ile-Ife, Nigeria.

### Study design

The study was a double-blind placebo-controlled randomised trial. Children aged 12–59 months were randomly assigned to receive either albendazole or placebo every four months for 12 months with a follow-up at 14 months. Albendazole and placebo tablets were identical and manufactured by GlaxoSmithKline. All eligible children received either treatment or placebo tablets on four occasions. Children were directly supervised to ensure that they took their tablet(s). Children aged one year received 200 mg of albendazole (1 tablet) and children aged ≥ 2 years received 400 mg (2 tablets) of albendazole [17]. Mothers were asked to inform the fieldworkers if their children had any adverse reactions to the anthelminthic drugs. Children in the placebo group were treated with albendazole at the end of the study. This trial is reported in accordance with the CONSORT guide-lines for randomised studies [19].

Prior to commencing the trial SOA placed the albendazole and placebo tablets in containers labelled either A or B. The treatment coordinator, SFM, oversaw the allocation of treatments to the children. During the first assessment each alternate child was assigned tablet B on returning a stool sample. Experienced physicians (TCA and Dr. OK Onabajo) enrolled all participants, measured all study endpoints, and were kept masked to treatment allocation of children. Field workers involved in data collection and mothers of participating children were also masked to the treatment allocation.

### Procedures

The primary outcome was prevalence and intensity of STHs. Children were screened for STHs at 0, 4, 8, 12, and 14 months. Stool samples were obtained before treatment was provided and stools were processed by formol-ether concentration [20]. STH infection was defined by the presence of eggs of *A. lumbricoides*, *T. trichiura*, hookworm or *Schistosoma haematobium *in the stool sample. An indirect measure of helminth intensity was obtained by counting eggs per gram of faeces (epg). Helminth intensity was categorised into light and moderate infections [11]. To maintain consistency all stool samples were examined by PK.

All children received 10 ml of multivitamins (over two days) as an incentive at each time point. Each 5 ml of multivitamin contained: Vitamin A 3000 IU, Vitamin B2 2.0 mg, Nicotinamide 15.0 mg, Vitamin B1 1.5 mg, Vitamin B6 2.0 mg, Vitamin D2 400 IU, D panthenol 1.0 mg.

### Statistical analysis

Estimation of sample size was based on the percentage change in malaria attacks for patients with *Ascaris *treated with albendazole. In a pilot study patients infected with helminths had more malaria attacks (11.1%) than patients who were not infected (4%) (P. Kirwan unpublished data). To detect a 7.1% reduction in malaria attacks, a study with 90% power and a significance level of P < 0.05 would need a sample size of 572 children. In the pilot study 42% of study participants did not return faecal samples. To account for this lack of compliance with faecal samples and a loss to follow-up (30%) the sample size was adjusted to 1055. The data on epg were assessed for normality visually and statistically. Epg values did not conform to a normal distribution and therefore the epg data were log transformed (epg +1) for the purposes of statistical analysis. Log transforming the epg data normalised the distribution of the values. All statistics were carried out in SPSS 14. Socio-economic status (SES) was calculated as a score based on the number of key possessions in a person's household.

A chi-square analysis was used to test the difference in the prevalence of *Ascaris *among age groups, villages and between the sexes at baseline. The influence of factors (age, village and sex) on epg were analysed by analysis of variance (ANOVA), using 3-way ANOVAs. In some cases least squares difference (LSD) *post-hoc *tests were applied to tease out the major sources of variation within factors.

Children that were lost to follow-up and children that were analysed were compared on the basis of their baseline characteristics, age, sex, village and SES and parasitic infections. A similar comparison was also undertaken to compare the characteristics of children in the treatment and placebo groups at inclusion into the study. Chi-square analysis was used to test proportions or Fisher's exact test when more than 20% of the cells had expected counts of less than five and 2-sample t-tests to test epg and SES.

The prevalence of *A. lumbricoides *was analysed using a linear mixed effects model using a logit function with the assumption that the observed data were binomially distributed. The analyses were run in R v2.6.2 [21] using the function *lmer *contained in the package *lme4*. A random effect for each individual was included to take account of the nested data structure (multiple observations made on each child). The effect of treatment (treatment or placebo) and age were included as fixed factors with time as a covariate. An interaction term between time and treatment was also included in the model. Parameter effects in the mixed effects model are presented as log(*odds*). In order to determine the proportional change in *odds *in the figures, we calculate exp(log(*odds*)).

Mean epg was measured over five time points from the same individuals. Since the data was not independent, it was analysed by repeated measures rmANOVA (General Linear Model) with the different time points as a within-subject factor. Group, age and sex were chosen as the between-subject factors; these factors were entered into the model if they were significant in the 3-way ANOVA. Since the data did not meet the requirements of sphericity, the Huynh-Feldt adjustment to the degrees of freedom was used to interpret the output on the side of caution.

## Results

**Figure 1:**
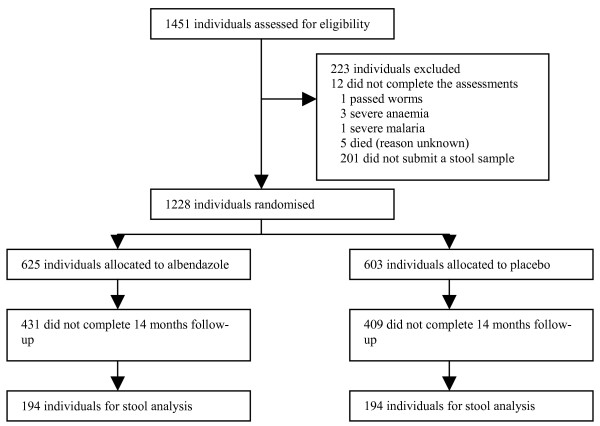
**Trial Profile**.

Figure 1 shows the trial profile. 1228 subjects were randomised into this study; 625 to albendazole and 603 to placebo. 31.6% of the eligible children complied with all the follow-up assessments.

### Prevalence and intensity of helminth infections at baseline

50.5% children were infected with any helminth at baseline (Table 1). *A. lumbricoides *was the predominant STH infection with 47.6% of the children being parasitised (Table 1). 3.7% of the children were infected with *T. trichiura*, 4.3% with hookworm and 1.1% with *S. haematobium *(detected in stool samples). The majority of *Ascaris *infections were of light intensity (85.9%); 14.1% of the children had moderate infections. All *T. trichiura *and hookworm infections were of light intensity.

**Table 1:** Baseline patterns of STH and *S. haematobium *infections in children aged 1–4 years.

		Age (years)			
	
	Overall (N = 1228)	1 (N = 422)	2 (N = 288)	3 (N = 280)	4 (N = 238)
Any helminth					
No. infected	620 (50.5%) ^a^	167 (39.6%)	161 (55.9%)	172 (61.4%)	120 (50.4%)
					
*A. lumbricoides*					
No. Infected	585 (47.6%)	161 (38.2%)	157 (54.5%)	159 (56.8%)	108 (45.4%)
Mean epg ± SE	1077 ± 78	504 ± 67	1386 ± 190	1636 ± 218	1059 ± 166
					
*T. trichiura*					
No. Infected	46 (3.7%)	6 (1.4%)	12 (4.2%)	15 (5.4%)	13 (5.5%)
Mean epg ± SE	4 ± 2	1 ± 1	4 ± 2	2 ± 1	12 ± 11
					
Hookworm					
No. Infected	53 (4.3%)	8 (1.9%)	10 (3.5%)	19 (6.8%)	16 (6.7%)
Mean epg ± SE	4 ± 1	2 ± 1	3 ± 2	7 ± 4	7 ± 3
					
*S. haematobium*					
No. Infected	14 (1.1%)	3 (0.7%)	1 (0.3%)	6 (2.1%)	4 (1.7%)
Mean epg ± SE^b^	0.03 ± 0.01	0.02 ± 0.01	0.01 ± 0.01	0.05 ± 0.02	0.05 ± 0.02

a % prevalence

b The maximum number of eggs detected in an individual was 6

The prevalence of *Ascaris *increased with age being 38.2% in children aged one year and 56.8% in children aged three years and this was statistically significantly (Table 1; χ^2 ^= 30.563, df = 3, P < 0.001). There was no statistically significant difference in the prevalence of *A. lumbricoides *between villages and sexes. Older children aged two (1386 ± S.E. 190), three (1636 ± S.E. 218) and four (1059 ± S.E. 166) years had a higher mean epg for *A. lumbricoides *than younger children aged one year (504 ± S.E. 67) (3-way ANOVA with village, age and sex as factors, model R^2^_adj _= 0.048, main effect of age, F_3, 1196 _= 16.333, P < 0.001). Females had a higher epg (1222 ± S.E.116) than males (941 ± S.E.107)(main effect of sex, F_1, 1196 _= 6.455, P = 0.011). There was no statistically significant difference in mean epg between villages. The LSD *post-hoc *analysis confirmed that mean epg was significantly different between children aged one year and two (P < 0.001), three (P < 0.001) and four years (P = 0.013); children aged two and four years (P = 0.008) and children aged three and four years (P = 0.003).

No adverse events were recorded concerning albendazole treatment. The baseline characteristics were similar for children who were analysed and who were lost to follow-up (Table 2). The randomised design resulted in a similar distribution of baseline variables between the treatment and placebo groups (Table 2).

**Table 2:** Baseline characteristics for individuals that were lost to follow-up and individuals that were analysed, and individuals in the treatment and placebo group

	Individuals lost to follow-up (N = 840)	Individuals analysed (N = 388)	P	Treatment Group (N = 194)	Placebo Group (N = 194)	P
**Characteristic**						
Age (months)						
12–23	287 (34.2%)	135 (34.8%)	0.336*	72 (37.1%)	63 (32.5%)	0.374*
24–35	187 (22.3%)	101 (26%)		46 23.7%)	55 (28.4%)	
36–47	194 (23.1%)	86 (22.2%)		39 (20.1%)	47 (24.2%)	
48–59	172 (20.5%)	66 (17%)		37 (19.1%)	29 (14.9%)	
Sex						
Male	443 (52.7%)	193 (49.7%)	0.329*	103 (53.1%)	90 (46.4%)	0.187*
Female	397 (47.3%)	195 (50.3%)		91 (46.9%)	104 (53.6%)	
Village						
Akinlalu	99 (11.8%)	86 (22.2%)	0.588*	43 (22.2%)	43 (22.2%)	0.562*
Ipetumodu	378 (45%)	157 (40.5%)		80 (41.2%)	77 (39.7%)	
Moro	160 (19%)	59 (15.2%)		33 (17%)	26 (13.4%)	
Edun-abon	203 (24.2%)	86 (22.2%)		38 (19.6%)	48 (24.7%)	
Socio-economic status						
Mean ± SE	6.49 ± 0.10	6.28 ± 0.06	0.077┼	6.39 ± 0.13	6.59 ± 0.15	0.324┼
						
**Parasitic infections**						
*Ascaris lumbricoides*	409 (48.7%)	176 (45.4%)	0.277*	88 (45.4%)	88 (45.4%)	1*
Mean epg ± SE	1060.69 ± 91.70	1111 ± 149.53	0.581┼	1095.19 ± 237.48	1126.81 ± 182.39	0.777┼
						
*Trichuris trichiura*	34 (4%)	12 (3.1%)	1§	5 (2.6%)	7 (3.6%)	0.558*
Mean epg ± SE	5.16 ± 3.07	1.70 ± 1.05		0.81 ± 0.47	2.6 ± 2.06	
						
Hookworm	38 (4.5%)	15 (3.9%)	0.598*	5 (2.6%)	10 (5.2%)	0.188*
Mean epg ± SE	4.86 ± 1.47	3.41 ± 1.75		3.56 ± 2.91	3.26 ± 1.97	
						
*Schistosoma haematobium*	7 (.8%)	7 (1.8%)	0.153§	3 (1.5%)	4 (2.1%)	1§
Mean epg ± SE	0.02 ± 0.01	0.06 ± 0.02		0.06 ± 0.04	0.06 ± 0.03	

* χ^2 ^test

┼ t-test

§ Fisher's exact test

### Effect of treatment on *A. lumbricoides *infection

**Figure 2:**
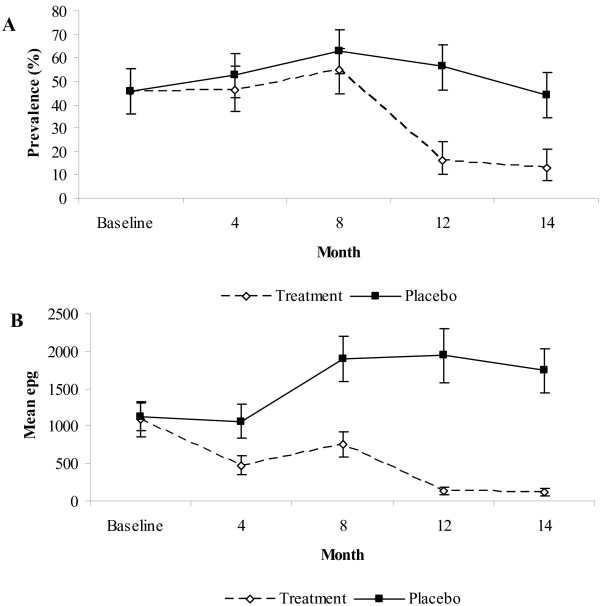
**(A) Prevalence rates ± C.I. (*A. lumbricoides*) and mean epg in treatment and placebo groups during the follow-up period**. (B) Mean epg ± S.E. Treatment group N = 194, Placebo group N = 194.

**Figure 3:**
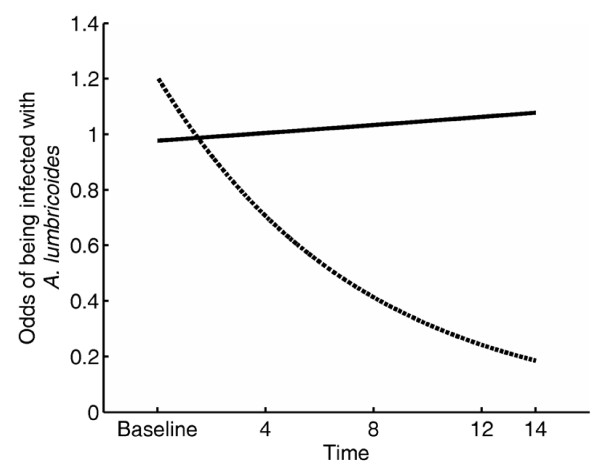
**The odds of testing positive for *A. lumbricoides *as a function of time for children in the treatment and placebo groups**. The placebo group is represented by the solid line while the treatment group is represented by the dotted line.

**Figure 4:**
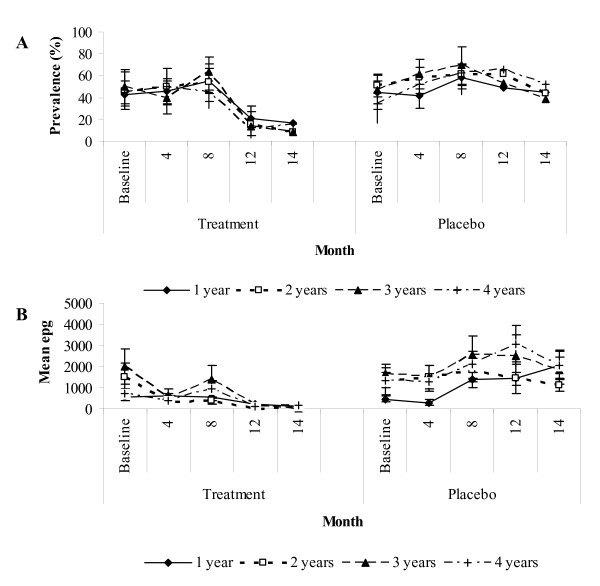
**(A) Prevalence rates ± C.I. (*A. lumbricoides*) and mean epg in treatment and placebo groups for each age group during the follow-up period**. (B) Mean epg ± S.E.

The prevalence of *A. lumbricoides *in the placebo group remained persistently high throughout the follow-up when compared to the treatment group (Figure 2A). It took three rounds of anthelminthic treatment before the prevalence of *A. lumbricoides *dropped in the treatment group. There was a significant interaction between treatment group and time in the linear mixed effects model analysis (P < 0.001; Table 3). At the beginning of the trial, the odds of being infected with *A. lumbricoides *was slightly larger in the treatment group, but not significantly so. However, as time progressed, the odds of being infected decreased in the treatment group (Table 3; Figure 3). In contrast, the placebo group had no significant relationship with time and had a constant odds of infection (Figure 3). Prevalence peaked at 8 months for both the treatment and placebo groups. By the end of the follow-up period (14 months), 12.9% of the children were infected with *A. lumbricoides *in the treatment group, while 43.8% were infected in the placebo group. The prevalence of *A. lumbricoides *was comparable for all age groups and demonstrated a similar pattern within the treatment and placebo groups over the study period (Figure 4A). The group*age interaction was not significant (P > 0.1) in the linear mixed effects model and was dropped from the final model.

**Table 3:** Linear mixed effects model for the prevalence of *A. lumbricoides *in children aged 1–4 years, with parameter estimates (expressed as log-odds) and associated standard error of the estimate and p-values.

Coefficient	Estimate	Std. Error	P-value
Intercept	-0.02	0.16	0.89
			
Group (Treatment)	0.21	0.19	0.274
			
Age (years)			
1	Reference	-	-
2	0.14	0.17	0.40
3	0.10	0.18	0.59
4	0.04	0.20	0.82
			
Time	0.01	0.01	0.59
			
Group(treatment):Time	-0.14	0.02	<0.001

The epg varied significantly over time (Figure 2B; rmANOVA, main effect of time (within subject analysis) F_3.7,1439.7 _= 21.93, P < 0.001). Unlike prevalence, when compared to the placebo group mean epg in the treatment group dropped after one round of anthelminthic treatment, rising slightly at 8 months but remaining at low levels throughout the follow-up period (Figure 2B). The mean epg in the placebo group increased after 4 months and stabilised (time*group interaction (within subject analysis) F_3.7,1439.7 _= 18.57, P < 0.001). The mean epg dropped by 89.3% in the treatment group from 1095 (S.E. 237) at baseline to 117 (S.E. 50) at 14 months, whereas the mean epg increased by 54.5% in the placebo group from 1126 (S.E. 182) at baseline to 1740 (S.E. 291) at 14 months (main effect of group (between subject analysis) F_1,382 _= 46.42, P < 0.001). At baseline, the number of moderate infections in the treatment and placebo group was 12 (6.2%) and 15 (7.7%) respectively. By the end of the follow-up the number of moderate infections dropped to 2 (1%) in the treatment group and increased to 23 (11.9%) in the placebo group.

The two- and three-way interactions with age and sex were non-significant (P > 0.1) and were therefore dropped from the final rmANOVA model. The pattern of epg across time was similar for the age groups (Figure 4B; time*age interaction (within subject analysis) F_11.3,1439.7 _= 2.11, P = 0.211). In the treatment group, epg decreased from baseline for children aged two to four years, increasing at 8 months and decreasing thereafter for all age groups. In comparison, in the placebo group the epg for children aged two to four years remained high at 4 months, increasing for all age groups at 8 months and continued to be high throughout the study period. The pattern of epg was similar for the age groups within the placebo and treatment groups over time, which was verified by the non-significant time*group*age interaction which was dropped from the final rmANOVA model. There was no significant main effect of age thus the age groups did not vary in their epg (Figure 4B; main effect of age (between subject analysis) F_3,382 _= 1.549, P = 0.202).

**Figure 5:**
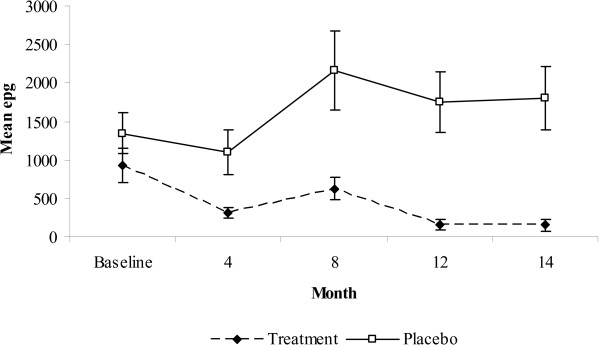
**Mean epg (± S.E.) (*A. lumbricoides*) in treated and placebo groups for females during the follow-up period**. Treatment group N = 91, Placebo group N = 104.

**Figure 6:**
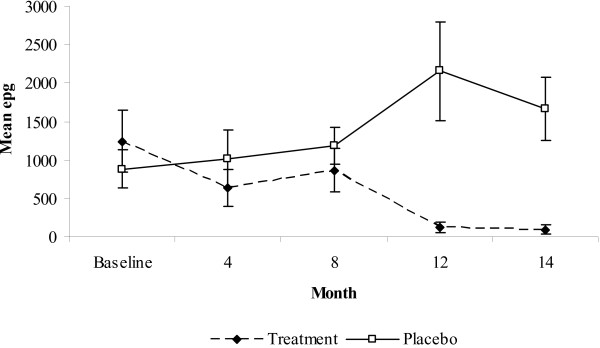
**Mean epg (± S.E.) (*A. lumbricoides*) in treated and placebo groups for males during the follow-up period**. Treatment group N = 103, Placebo group N = 90.

The pattern of epg across time was similar for the sexes (Figures 5 and 6; time*sex interaction (within subject analysis) F_3.7,1439.7 _= 1.027, P = 0.389). For females the mean epg decreases after 4 months in the treatment group and remains at low levels throughout the follow-up period when compared to the placebo group where mean epg remains higher with a sharp increase at 4 months (Figure 5). In contrast to females, although mean epg for males decreases after 4 months in the treatment group, the mean epg for both treatment and placebo groups does not diverge until 8 months i.e. after three rounds of treatments. Despite this, the pattern of epg was similar for the sexes within the treatment and placebo groups over time, this was demonstrated by the non-significant interaction between time*group*sex which was dropped from the final rmANOVA model. There was a significant main effect of sex so the sexes varied in their epg (Figures 5 and 6; main effect of sex (between subject analysis) F_1,382 _= 4.606, P < 0.032).

## Discussion

The results presented here revealed that 50% of the preschool children in these semi-urban communities are infected by one or more helminths, the most prevalent STH being *A. lumbricoides *(47.6%). Our study demonstrated that repeated four-monthly anthelminthic treatments with albendazole were successful in reducing prevalence and intensity of *A. lumbricoides *infections in preschool children.

The study may have some potential limitations that need to be considered when interpreting the findings. Participants were not randomly selected from the community. Achieving a random sample of subjects in this field setting would prove very difficult owing to the widely dispersed nature of these semi-urban communities, and to the restricted age group being studied. Non-random selection of subjects is common in epidemiology studies [22,23] and we believe that the moderate sample size, small age range and randomised design compensate for the non-random selection of subjects. 68.4% of children were lost to follow-up. This high attrition rate may be attributed to the difficulty in accessing such young children particularly because they are not of school-going age. School-based studies provide a better infrastructure and can improve compliance considerably[24]. Bias caused by such losses was probably minor because those lost were similar with respect to important baseline characteristics to those that were analysed. Epg was used as an indirect measure of prevalence and intensity of helminth infections. Getting a true measure of worm burden by expelling the worms from the gut is very difficult, time-consuming work and is not feasible for such large-scale studies [25].

The predominant infection in these communities was *A. lumbricoides *but lower prevalences of *T. trichiura*, hookworm and *S. haematobium *were also detected. The prevalence of *A. lumbricoides *increased with age. Maximum prevalence values for *A. lumbricoides *are usually observed when children are 5–10 years old [8]. A previous study in this region of Nigeria demonstrated the prevalence of *A. lumbricoides *to be 88.5% in school children aged 5–15 years [9]. Higher prevalences of *Ascaris *in studies on children aged 0–48 months, have been found in China (80%) [26], the Philippines (77%) [27], and Sri Lanka (62%) [28]. Prevalence can vary geographically, lower prevalences have been found in children aged 12–48 months in Zanzibar (41.5%) [29], and children aged 12–47 months in Nigeria (20.5%) [30].

The majority of *Ascaris *infections in this study were of light intensity (85.9%), few children harboured moderate intensity infections (14.1%). The intensity of infection increased with age, children aged one year had a lower mean epg than older children. Children aged 5–15 years usually have heavier worm burdens [31]. A Nigerian study that examined strategies for community control of *Ascaris*, showed that intensity peaked in children aged 10–14 years (mean epg >20,000) and declined thereafter in older children and adults (mean epg <10,000) [18]. In the present study, females had a higher mean epg. Other studies have also demonstrated that females have heavier worm burdens [9,32,33] but the cause of this is yet unknown [8] and could be related to behavioural differences between males and females. A study in South African school children showed that female children were more likely to be infected with *Ascaris *and also had a higher incidence of soil-eating than males [34].

Four-monthly anthelminthic treatments with albendazole were effective in reducing the prevalence and intensity of *A. lumbricoides *infections. The rmANOVA analysis demonstrated that there was a statistically significant difference in epg between treatment and placebo groups. Unlike prevalence, the intensity of *A. lumbricoides *infections dropped after one round of treatment. Prevalence is regarded as a relatively insensitive measure of reinfection because of the aggregated distribution of worms per child [9]. It has been well documented that marked changes in intensity may be accompanied by only small changes in prevalence [35-37]. Furthermore, because of high rates of reinfection in endemic regions the aim of deworming is not to reduce prevalence but to reduce morbidity by decreasing worm burden. Early and regular administration of single-dose anthelminthic drugs recommended by WHO reduces the occurance, extent, severity and long-term consequences of morbidity, and in certain epidemiological conditions contributes to sustained reduction in transmission [38].

Holland and colleagues [39] proved that four-monthly anthelminthic treatments with levamisole were better than six-monthly or one-yearly treatments in reducing prevalence and intensity of *A. lumbricoides *infection in children aged 5–15 years. In the present study, the drop in prevalence (33%) of *Ascaris *between baseline and 14 months was lower than the result demonstrated by Holland et al. [39] for four-monthly treatments where there was a 54% drop in prevalence by the end of the 13 month study period. The significant reduction of mean epg (89%) in the treatment group at the end of the present study was comparable to the reduction of *Ascaris *intensity shown in Holland's study (92.4%; taken from graph). A study in an urban community in Malaysia showed that six-monthly treatments with albendazole were effective in reducing prevalence and intensity of *A. lumbricoides *infection [36]. After 12 months (two six-monthly treatments) the prevalence was reduced by 12% in treated children aged two to six years (prevalences were taken from a graph in Chan et al. [36]). This suggests that four-monthly treatments may be better in reducing prevalence of *Ascaris *in a similar age group. Mean worm burden was reduced from 3.2 to 1.9 worms in the two to six year olds. Since the analysis for this present study is based on an indirect measure of worm burden, epg, it is difficult to compare these results.

A study examining the effect of iron supplementation and anthelminthic treatment on motor and language development of preschool children aged 0.5–5 years in Zanzibar used repeated three-monthly treatments with mebendazole [29]. After 12 months, prevalence of *Ascaris *dropped by 22.3% in the treatment group when compared to the placebo group. Results from the present study showed that four-monthly treatments with albendazole reduced the prevalence of *A. lumbricoides *by 40.2% in the treatment group after 12 months. Although these studies took place in different epidemiological settings, the data presented here suggest that four-monthly treatments with albendazole were more effective than three-monthly treatments with mebendazole. Three-monthly treatments significantly reduced *Ascaris *intensity [29]; comparing this data with results presented here is difficult as the intensity data is presented in geometric means. In contrast to the work carried out by Stoltzfus et al. [29] a study by Thein-Hlaing et al. [40] showed that three-monthly anthelminthic treatments were better than four-monthly treatments at reducing the prevalence of *A. lumbricoides*; however this Burmese study was undertaken in children and adults. The pre-treatment prevalences for all ages and children less than 15 years were 83.6% and 77.1%, respectively. After two years of three-monthly treatments the corresponding prevalences fell to 21% and 5% respectively.

In contrast to the results presented here, a study in 117 Bangladeshi preschool children aged 2–6 years showed that two-monthly anthelminthic treatments were better than four-monthly treatments in reducing the prevalence of *A. lumbricoides *infections [41]. After one round of treatment, prevalence in the treatment group dropped from 78% to 8% and remained high in the placebo group increasing from 71% to 74%. After 12 months, the mean worm burden in the placebo group was 4.1 (maximum: 40) for infected children. An examination of the nine infected children in the treatment group, yielded five children with small immature worms and four children with one adult worm. Another study in Bangladesh, used two-monthly repeated treatments with albendazole in 1402 children aged 2–6 years old [42]. When assessing the effectiveness of anthelminthic treatment on prevalence of STH infections, a random sub-sample of children were screened eight months after the initial treatment. Prevalence of *Ascaris *in the treatment group fell by 73% while prevalence in the placebo group fell by 2%. Similarly, a study in Madagascar, examining helminth-malaria co-infections in children and adults used two-monthly treatments with levamisole [43]. Prevalence and intensity of *A. lumbricoides *collapsed immediately in the treatment group after the first round of treatment. Prevalence and intensity remained high in the placebo group throughout the study period. While shorter deworming treatment intervals of two and three months may be better than four-monthly intervals at reducing prevalence of *A. lumbricoides*, our results demonstrate that deworming children at four-monthly intervals are just as effective in the immediate reduction of intensity.

In 2002 WHO organised an informal consultation to assess previous recommendations to avoid the use of anthelminthic drugs in children less than 24 months [17]. The consultation concluded that although there is little published information about the use of anthelminthic drugs in this age group, the data that exists offers no obvious reason for excluding children of this age group from treatment. They recommended a 200 mg dose of albendazole instead of 400 mg to be given to children aged less than 24 months. The present study has demonstrated that four-monthly treatments were effective in the reducing the prevalence and intensity of *Ascaris *infections in children aged one year. Future studies should investigate the efficacy of 200 mg anthelminthic treatments for children aged one year and determine the optimal dose of albendazole to be used in this age group.

## Conclusion

Results from this study show that the moderate prevalence and low intensity of STH infection in these preschool children necessitate systematic treatment of these children in child health programmes [11]. Although nearly 20 years have passed since the study by Holland et al [39] was conducted, STHs still remain a significant problem in this region and will most likely continue to do so in the future without proper intervention.

Repeated four-monthly deworming treatments were effective in reducing prevalence and more importantly intensity of *A. lumbricoides *infections with 1% and 11% of individuals harbouring moderate infections at the end of the study in the treatment and placebo groups respectively. While shorter treatment intervals may be better at reducing prevalence of *Ascaris *infections, in reality, two- and three-monthly treatments may not be feasible and in the long term, could promote drug resistance rendering this broad spectrum anthelminthic less effective against STH infections.

Overall, this study has clearly demonstrated the need to incorporate preschool children into deworming programmes in endemic regions and to investigate innovative ways of delivering cost effective deworming treatment to this high risk age group.

## Competing interests

GlaxoSmithKline sponsored the drug albendazole which was used in the study. The authors declare that they have no competing interests. The authors also declare that they have no financial competing interests.

## Authors' contributions

CVH conceived the study and designed it with PK with input from all the other authors. SOA and TCA chose the villages and provided input for the logistics of fieldwork. PK conducted the fieldwork with SM and contributions from the other authors. PK did the stool analysis, carried out statistical analysis with ALJ and input from CVH, and drafted the manuscript. All authors contributed to the final version of the manuscript and read and approved it.

## Pre-publication history

The pre-publication history for this paper can be accessed here:

http://www.biomedcentral.com/1471-2334/9/20/prepub
